# A Facile Method to Prepare a Superhydrophobic Magnesium Alloy Surface

**DOI:** 10.3390/ma13184007

**Published:** 2020-09-10

**Authors:** Jiyuan Zhu, Haojie Jia

**Affiliations:** College of Mechanical and Control Engineering, Guilin University of Technology, Guilin 541004, China; 18656959510@163.com

**Keywords:** superhydrophobic, wettability, thermal stability, corrosion resistance

## Abstract

The application of superhydrophobic materials has been handicapped by complex processes and poor environmental friendliness. Magnesium alloys are widely used in daily production due to their low density and good casting properties. A facile and environmentally friendly method was proposed to prepare a superhydrophobic layer with coral-like microstructure on the surface of AZ91D magnesium alloy by high temperature heating. The prepared superhydrophobic surface has a contact angle of 159.1° and a rolling angle of 4.8°. The corrosion current of superhydrophobic surface has been reduced by about two orders of magnitude relative to the magnesium alloy substrate and its inhibition efficiency is 96.94%, which demonstrates its great corrosion resistance. In addition, the superhydrophobic surface has great thermal stability. When the temperature rises to 190 °C, the contact is still above 150°. Excellent self-cleaning and advantages in preparation efficiency, environmental protection and cost-effectiveness will boost its good application prospects.

## 1. Introduction

Found on many animals and plants, such as lotus leaf surface, mosquito’s eyes, and water strider’s legs [1,2,3], superhydrophobicity is a common phenomenon in nature. Material surfaces, with a water contact angle greater than 150° and a rolling angle less than 10°, are usually called superhydrophobic surfaces [4]. Superhydrophobic surfaces are gaining more and more research attention due to their great properties of anticorrosion [5,6,7], drag reduction [8,9,10], self-cleaning [11,12] and anti-icing [13]. Many methods to prepare superhydrophobic surfaces have been investigated, which mainly include chemical etching [14], electrodeposition [15], laser engraving [16], anodizing [17,18] etc. The excellent performance of superhydrophobic surfaces offer potential applications in many fields, such as microfluidic control [19], corrosion inhibition [20], nondestructive transportation [21], drag reduction [22], and so on. Superhydrophobic technology can be applied on the metal surface to slow down the corrosion rate of the metal surface to a certain extent [23].

Magnesium, and its alloys, are widely used in automobiles, aerospace parts, medical treatment and electronic communication due to its good ductility and conductivity, excellent specific stiffness and strength, and other advantages [24,25,26,27]. However, the low standard potential, poor corrosion resistance and high chemical activity have limited its application to a certain extent [28,29]. Surface treatment [30] has been an important attempt to improve the properties of magnesium alloy. The inherent water-repellent properties of superhydrophobic surfaces can reduce the exchange rate of metal ions and the electrochemical oxidation rate, thus greatly improving the corrosion resistance of metals and alloys [31]. Therefore, superhydrophobic preparation on the surface of magnesium alloy can effectively protect its surface against corrosion and broaden the use of magnesium alloy. Bao Yin et al. [32] etched magnesium alloy with nitric acid and a cupric nitrate mixed solution, and then modified it with a low surface energy solution and prepared the superhydrophobic surface with a contact angle of 162.1°. Liu et al. [33] prepared the superhydrophobic surface of magnesium alloy with excellent durability. Liu et al. [34] prepared the superhydrophobic surface of magnesium alloy with excellent corrosion resistance on AZ31 magnesium alloy by micro arc oxidation and immersion in stearic acid ethanol solution. Kang et al. [35] electrodeposited AZ31 superhydrophobic magnesium alloy with good self-cleaning properties in the mixed solution of ceric nitrate and stearic acid ethanol. Wu [36] et al. prepared superhydrophobic films with regular hexagon microstructure and excellent friction resistance on the surface of magnesium alloy. Liu et al. [37] prepared inorganic organic hybrid superhydrophobic films with pH-sensitive "core-shell" nanostructure inhibitors on AZ61 magnesium alloy by dip-coating method. However, these preparation methods are complex in technology [33,36], poor in environmental friendliness [32] and high in research cost [34], which are not conducive to the practical application of magnesium alloy. Therefore, it is of great value to study the preparation of superhydrophobic and anticorrosive surfaces of magnesium alloy with low cost and risk.

In this study, the surface of superhydrophobic magnesium alloy (SMA) was prepared by stearic acid treatment after stable heating of magnesium alloy sheet in a muffle furnace. The prepared super-hydrophobic layer has good corrosion protection performance to the magnesium alloy substrate. The samples show excellent self-cleaning and high temperature resistance. After high temperature heating, it can still maintain good superhydrophobic properties, which greatly broadens the application scope and service life of magnesium alloy. This preparation method does not need expensive equipment, complicated preparation processes, and substances such as fluoride and strong acid and alkali that cause hazards to the environment. It has excellent environmental performance, economy and high efficiency. At present, there are few articles about the preparation of superhydrophobic surfaces by directly heating the substrate to form a microstructure. This method is a new attempt to prepare a superhydrophobic surface of magnesium alloy. All these advantages will boost its good application prospects.

## 2. Materials and Methods

### 2.1. Materials

The 15 mm × 15 mm × 3 mm AZ91D magnesium alloys (8.5–9.5% Al, 0.45–0.9% Zn, 0.17–0.4% Mn) were used as material and purchased via the Hebei Tengshi metal materials Co., Ltd. (Xingtai, China). Stearic acid (SA) and sodium chloride (NaCl) were supplied by Xilong Scientific Co., Ltd. (AR, Shantou, China). Anhydrous ethanol was supplied by Fuyu Fine Chemical Co., Ltd. (Tianjin, China). 

### 2.2. Methods

The AZ91D magnesium alloy was successively grounded with SiC papers from 600 to 1200 grit until it shone. After this, it was put into anhydrous ethanol and deionized water for ultrasonic cleaning for 10 min to remove the oil and impurities. Then, 0.3 g of stearic acid solid particles and 29.7 g of anhydrous ethanol were added into a 50 mL beaker to prepare 30 g of 1 wt.% ethanol stearic acid solution.

The pre-treated magnesium alloy (MA) substrates were put into a muffle furnace (KSL-1700X, Hefei kejing Material Technology Co., Ltd, Hefei, China) with the heat holding temperatures of 365 °C, 375 °C, 385 °C and 395 °C, and the magnesium alloy was covered with a crucible to prevent irreversible effects on the surface structure caused by the fast temperature rise. The heating time was set as 100 min, and the heat holding time was set as 30 min, 60 min, 90 min and 120min, respectively. After heating, the MA were removed and cooled to room temperature. Subsequently, the MA was soaked in a pre-prepared solution of ethanol and stearic acid for 10 min. Finally, the MA was removed and dried in the air.

### 2.3. Sample Characterization

The morphologies and chemical composition of the samples were characterized by field emission scanning electronic microscopy (SEM, SU5000, HITACHI, 5.0 KV, Tokyo, Japan) quipped with an energy dispersive X-ray spectrometer (EDS, 15 KV, xflash6110, Bruker, Leipzig, Germany). The phase structure of the samples was studied with an X-ray diffractometry (XRD, smartlab9, Rigaku Corporation; Target: CuKa; 40 KV, 150 mA; Wavelength: 1.54056, Tokyo, Japan). The chemical compositions and valence states of the samples were measured using X-ray photoelectronspectroscopy (XPS, 250Xi, Thermo scientific, Waltham, Massachusetts, America) with the Al Kα X-raysource (hν = 1486.6 eV). The chemical compositions were also determined by Fourier transform infrared spectroscopy (FTIR, IRAffinity-1S, Shimadzu Corporation, 4000–450 cm^−1^, Tokyo, Japan). The static contact angle (CA) and sliding angle (SA) were measured by a contact angle measuring instrument (SDC-200, Sindin Precision Instrument Co., Ltd., Dongguan, China). Average contact angle measurements were obtained at 4 different locations using 8 μL droplets (deionized water). All electrochemical measurements were performed using a CS2350H electrochemical workstation (Wuhan Corrtest Instruments Corp., Ltd, Wuhan, China) using a conventional three electrode set-up with a platinum plate as the auxiliary electrode, a Ag/AgCl (Saturated KCl) electrode as the reference electrode, and the sample to be tested as the test electrode. The exposure area of the tested sample was 1 cm^2^ and the tests were done at room temperature. Polarization curves were obtained in 3.5 wt% NaCl solution. The measured potential was from −0.5V to 1.5 v vs. open circuit potential at a scan rate of 1 mVs^−1^. Before the test, the sample was in contact with the solution for 30 min to ensure the stability of the sample surface.

## 3. Results and Discussion

### 3.1. Wettability

**Figure 1:**
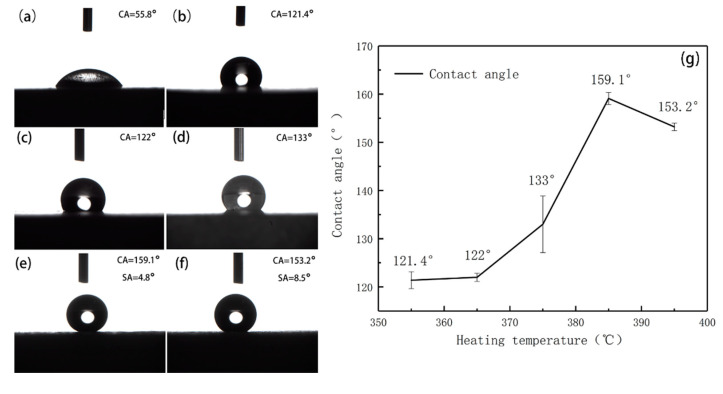
Contact angle images of the magnesium substrate (**a**) and samples with different heating temperatures: (**b**) 355 °C, (**c**) 365 °C, (**d**) 375 °C, (**e**) 385 °C, (**f**) 395 °C; Contact angle change trend (**g**).

Temperature is a critical factor affecting the growth of coral-like microstructures on the surface of magnesium alloy in this experiment. In order to prepare a SMA surface with excellent properties, the effect of heating temperature on surface wettability of MA is studied. The influences of different heating temperatures, 355 °C, 365 °C, 375 °C, 385°C and 395 °C, on the contact angle of the superhydrophobic surface are shown in the Figure 1. after 60 min in heat preservation. 

It can be seen from Figure 1a that the surface of the magnesium alloy gains the hydrophobic property when the heating temperature is 365 °C. As it increases to 375 °C, the contact angle gradually increases to 133°, as shown in Figure 1b. In Figure 1c, when the heating temperature increases to 385 °C, the static water contact angle of the droplets on the magnesium alloy surface reaches 159.1°. As the temperature continues to increase to 395 °C, the contact angle decreases to 153.2° in Figure 1d. When the heating temperature is 405 °C, the MA melts into powder. Therefore, the optimal heating temperature is 385 °C when the holding time is 60 min.

**Figure 2:**
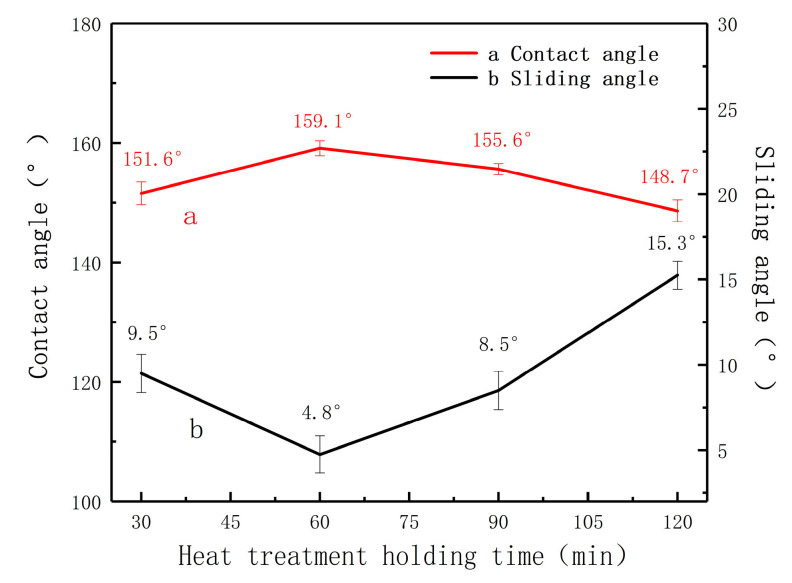
Contact angle of the samples with different heat holding time (385 °C): 30 min, 60 min, 90 min and 120 min.

The effect of heat treatment holding time on the surface wettability of MA is studied. Under the same heating temperature of 385 °C, the influences of the heat holding time on the contact angle of the MA surface are shown in the Figure 2.

It can be seen from Figure 2 that the static contact angle of water droplets on the magnesium alloy surface increases with the increase in heat holding time, and it reaches 159.1° when the heat treatment time is 60 min. As the heat holding time continues to grow, the contact angle gradually decreases.

The investigation shows that the water contact angle of magnesium alloy changes relatively little under the same heating temperature and different heat holding times; however, the contact angle of magnesium alloy varies greatly with the constant heat holding time and different heating times. Therefore, the heating time is one of the important factors affecting the wettability of the magnesium alloy surface. At the same time, a heating temperature of 385 °C and a heat holding time of 60 min are the best conditions for this experiment.

### 3.2. Surface Morphology

**Figure 3:**
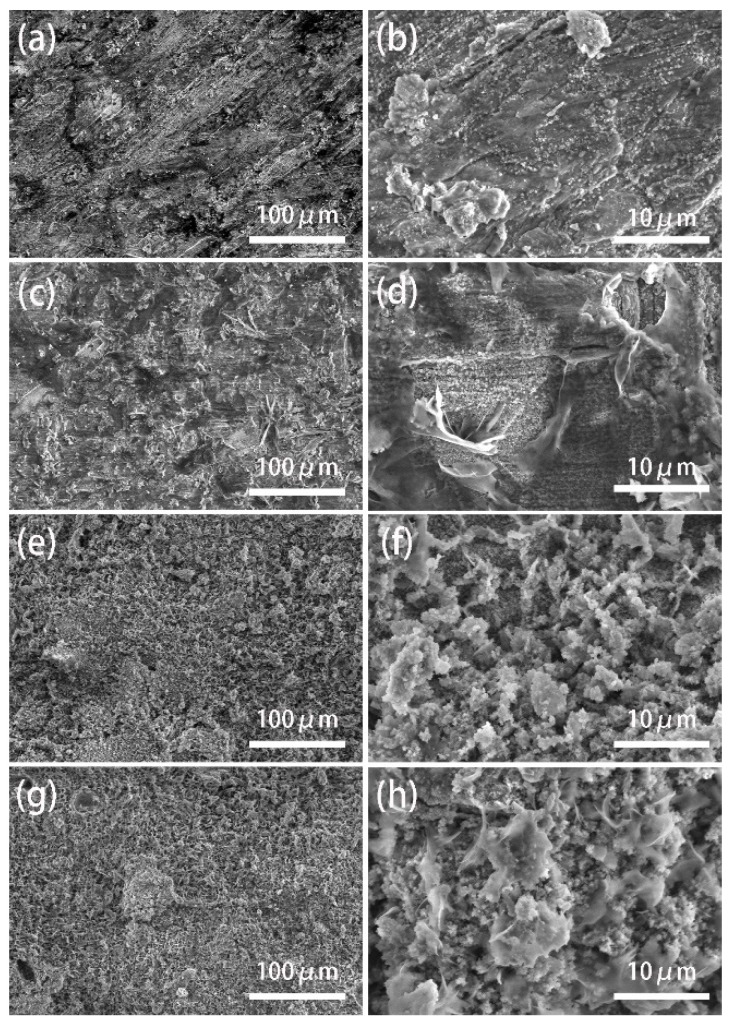
SEM images of the sample surfaces with different heating temperature: (**a**,**b**) 365 °C; (**c**,**d**) 375 °C; (**e**,**f**) 385 °C; (**g**,**h**) 395 °C.

To investigate the significant difference in wettability between the SMA surface and the untreated magnesium alloy, we compare the scanning electron microscope (SEM) images of samples processed in different conditions. As can be seen in Figure 3a,b, the surface is relatively flat and a sparse small microstructure begins to grow under 365 °C. At 375 °C, it can be observed in Figure 3c,d that more microstructures begin to grow on the surface of the magnesium alloy and the wettability gradually improves. As the temperature rises to 385 °C, it can be observed in Figure 3e,f that a layer of dense coral-like microstructure is formed on the magnesium alloy surface. The existence of pores in this structure, like the Cassie model, makes the air become trapped between grains when droplets contact the surface, which enhances the hydrophobicity of the material surface [2,38]. At 395 °C, it can be seen from Figure 3g,h that a considerable part of the original structure similar to the Cassie model is broken, leaving fewer microstructures to trap the air and prevent the wetting of the droplets, which is one of the causes of the decline of its wettability [39].

### 3.3. Surface Composition Analysis

In this section, the superhydrophobic surface is all prepared at a heating temperature of 385 °C and a holding time of 60 min.

**Figure 4:**
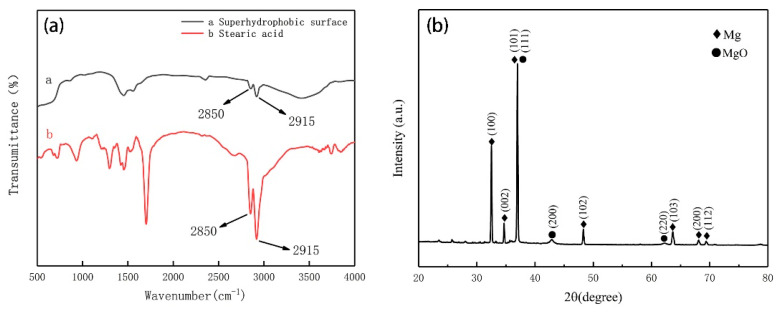
(**a**) FT-IR infrared spectra of the superhydrophobic magnesium alloy (SMA) surface and stearic acid, and (**b**) XRD diffraction patterns.

Figure 4a shows the FT-IR spectra of the superhydrophobic surface and stearic acid. It can be seen from the figure that, after the modification of stearic acid, absorption peaks exist at the wavelengths of 2850 cm^−1^ and 2915 cm^−1^ corresponding to the symmetric and antisymmetric methylene stretching vibrations, respectively, and this indicates the presence of hydrophobic long-chain alkyl on the sample surface [40,41,42]. Results demonstrate that stearic acid has been successfully bonded to the surface of the magnesium alloy substrate.

The XRD patterns of samples are illustrated in Figure 4b. According to the JADE (software, JADE6, Jade company, New Zealand) peaks, the XRD results show that the SMA displays characteristic peaks at 2θ of 32.5°, 34.7°, 36.9°, 48.3°, 63.7°, 68.1° and 69.4°, which are attributed to the (100), (002), (101), (102), (103), (200) and (112) crystalline planes of Mg [43]. In addition, the SMA displays characteristic peaks at 2θ 36.9°,42.9° and 62.3°, which are attributed to the (111), (200) and (220) crystalline planes of MgO [44].

**Figure 5:**
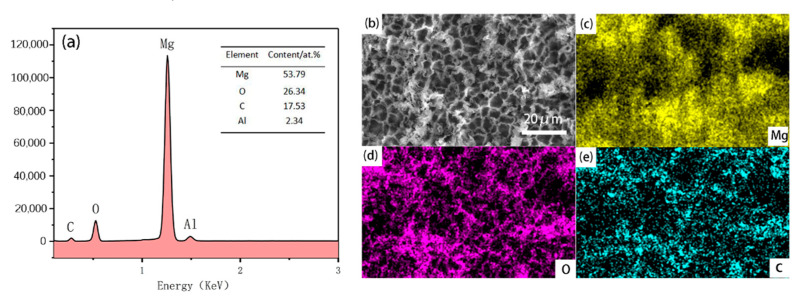
(**a**) Results of EDS on the SMA surface; (**b**) SEM morphology; (**c–e**) EDS elemental mapping images of the SMA surface.

We use EDS to help analyze the elemental composition of the SMA surface. Figure 5a shows the results of the superhydrophobic magnesium alloy (SMA) that was heated at 385 °C and kept warm for 60 min. The SEM surface morphology is shown in Figure 5b. As shown in Figure 5c–e, the Mg, O and C elements uniformly distribute on the surface of the SMA.

**Figure 6:**
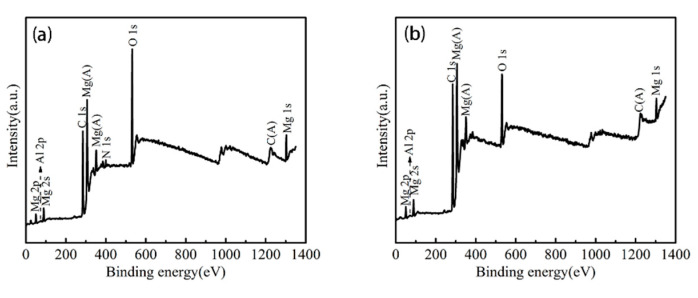
XPS survey spectrum of (**a**) magnesium alloy (MA), (**b**) superhydrophobic magnesium alloy (SMA).

To further determine the chemical compositions of the surface, an XPS test is conducted. Figure 6 shows the XPS survey spectrum of MA and SMA. It can be seen from the Figure 6a,b that the Mg, Al, O, C elements exist on the surface of MA and SMA. The possible reasons for the disappearance of the N element are as follows. First, the very small amount of N element on the superhydrophobic surface is blocked by the precipitation of Mg and the formation of MgO. Second, the AlN originally present on the surface of the magnesium alloy may react to form Al_2_O_3_ and N_2_ after high-temperature heating treatment.

**Figure 7:**
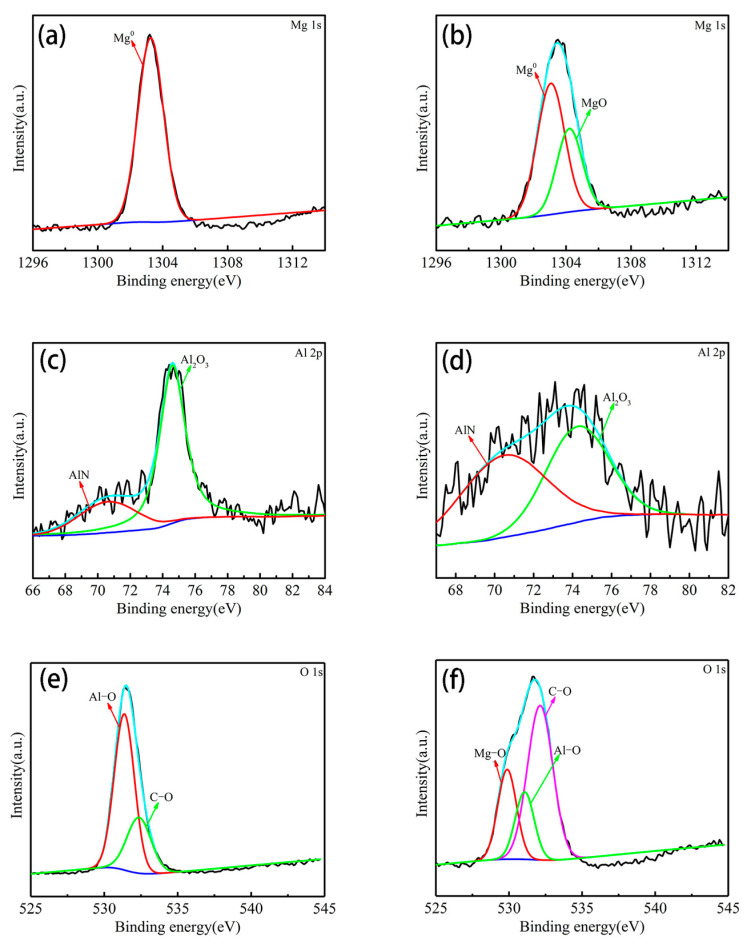
XPS high resolution spectrum of (**a**) Mg 1s, (**c**) Al 2p; (**e**) O 1s core level spectrum of MA, (**b**) Mg 1s and (**d**) Al 2p; (**f**) O 1s core level spectrum of SMA.

The detailed surface composition and chemical state, evaluated using high resolution spectra, were acquired for individual elemental peaks and deconvoluted using XPS peak software. As shown in Figure 7a, the deconvoluted Mg 1s core level peak consists of the magnesium (1303.3 eV). The deconvoluted Mg 1s core level peak (Figure 7b), consists of the Mg and MgO corresponding to 1303.3, 1304.2 eV, respectively. The deconvoluted Al 2p peak indicates the presence of Al_2_O_3_ (74.7 eV), AlN (70.3 eV) bonds in Figure 7c and Al_2_O_3_ (74.4 eV), AlN (70.6 eV) in Figure 7d. AlN may be an impurity. In sure 7e, the peaks at 531.4 and 532.3 eV can be assigned to Al–O and C–O. In Figure 7f, the peaks at 529.9, 531.2, 532.1 eV can be assigned to Mg–O, Al–O and C–O, respectively. These indicate that MgO is mainly formed during the reaction.

### 3.4. Corrosion Behavior

**Figure 8:**
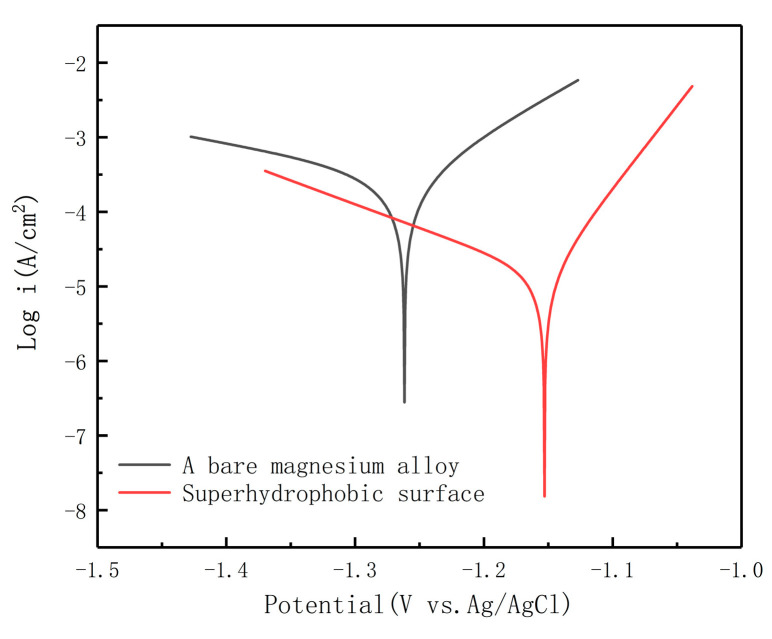
Potentiodynamic polarization curves of the AZ91D Mg substrate and the superhydrophobic surface in the 3.5 wt. % NaCl solution.

The potentiodynamic polarization curves of both bare and superhydrophobic magnesium alloys are presented in Figure 8.

In order to confirm the reliability of the electrochemical parameters, the polarization tests were performed in three times and the average values were obtained. Table 1 shows the corrosion current density (I_corr_) and corrosion potential (E_corr_), which were determined using the extrapolation method. Compared with the untreated AZ91D magnesium alloy surface, the corrosion potential of the SMA surface changes to a positive direction (from −1.2525 V vs. Ag/AgCl to −1.1495 V vs. Ag/AgCl), and the corrosion current density decreases by almost two orders of magnitude (from 3.1713 × 10^−4^ A·cm^−2^ to 9.7053 × 10^−6^ A·cm^−2^). The presence of the superhydrophobic coating further blocks the contact between the sodium chloride solution and the magnesium alloy substrate. This makes the superhydrophobic surface relatively difficult to be corroded, which may be one of the reasons for the positive shift of corrosion potential. In this table, the corrosion efficiency of the inhibitor (η) [45], which is calculated via the following Equation (1), is also provided.

(1) $$\eta =\frac{i^{0}{}_{corr}-i_{corr}}{i^{0}{}_{corr}}\times 100\%$$

where $i^{0}{}_{corr}$ and $i_{corr}$ are corrosion current densities of MA and SMA, respectively. According to the calculation, the inhibition efficiency of the SMA is 96.94%. The results show that the superhydrophobic layer can effectively enhance the corrosion resistance of the substrate.

### 3.5. Self-Cleaning Effect

**Figure 9:**
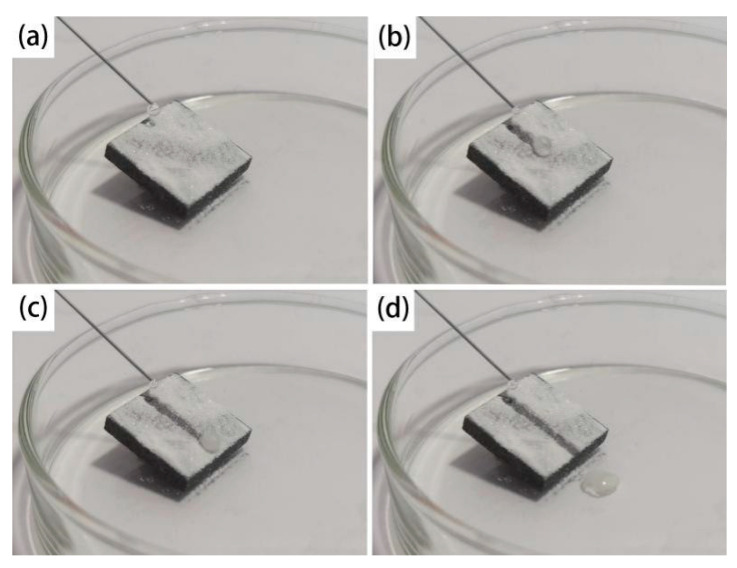
Self-cleaning performance of the SMA surface. (**a**) Dripping; (**b**) Water drop contact; (**c**) Water drop rolling; (**d**) Water droplets roll out.

To test the self-cleaning properties of a sample, water was dropped on the surface covered with fine sand as shown in Figure 9a. As a water droplet dripped onto the surface of the sample, under the action of surface tension, the water droplet and fine sand mixed and formed a cloudy droplet, which continued to roll until it fell off the surface as depicted in Figure 9b–d. The angle between the sample and the plane was about 12 degrees during testing. This angle was greater than the rolling angle on the surface of the SMA, so the droplet rolled down smoothly. The result indicates that the surface of the film modified by stearic acid has low adhesion and good self-cleaning performance.

### 3.6. Bouncing Performance Test

**Figure 10:**
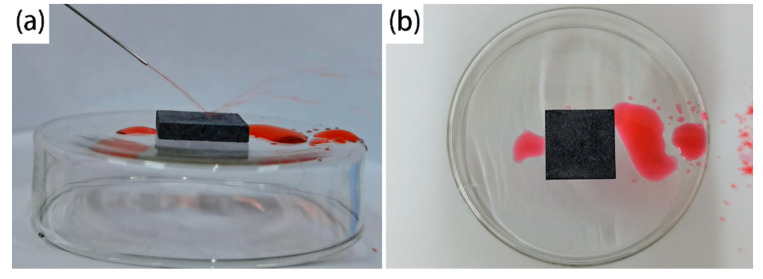
Bouncing performance of the SMA surface. (**a**) Red ink bouncing; (**b**) No residual liquid on the surface.

The red ink flow from the syringe was utilized to test the bouncing performance of the SMA surface. The water flowed out of the syringe and fell on the surface of the sample. The water rebounded on the surface of the sample and popped out to the other side. Figure 10a,b show the whole process. After the test, there was no liquid residue on the surface, which shows the excellent water repellency of the SMA surface.

### 3.7. Thermal Stability

**Figure 11:**
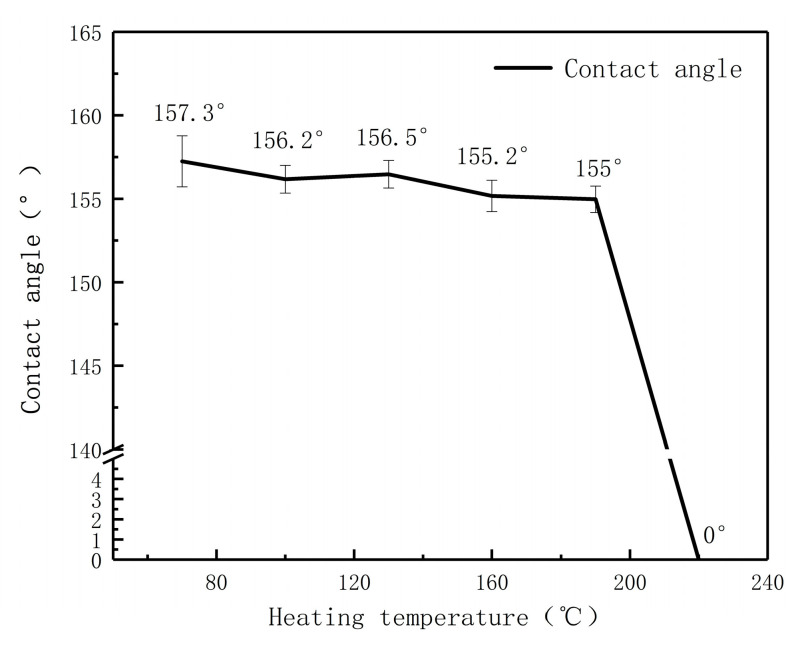
Thermal stability test of the SMA.

The thermal stability experiment of the superhydrophobic surface is of great significance in practical application, especially when magnesium alloy parts are used at an elevated temperature. Figure 11 shows the change trend of contact angle of the SMA surface heated continuously for one hour at different temperatures.

The figure shows that the contact angle of the SMA surface decreases with the increase in temperature, and the contact angle remains above 150° when the continuous heating temperature is 190 °C. When the temperature rises to 220 °C, the surface of the sample changes into the superhydrophilic state. According to the research, the stearic acid molecular chains gradually break down, carbonize and decompose at 160 °C [46]. However, due to the fine microstructure on the surface of the SMA, its decomposition rate is slowed down. When the temperature rises to 220 °C, the molecular chains completely break, carbonize and decompose, which is the main reason for its transformation from superhydrophobic to superhydrophobic. Soaked in stearic acid ethanol solution for 10 min, the surface contact angle reaches 152.9° again and it changes to the superhydrophobic state.

## 4. Conclusions

In this experiment, AZ91D is heated at high temperature and modified with stearic acid to prepare SMA. The contact angle and rolling angle are 159.1° and 4.8°, respectively. The SMA surface has excellent superhydrophobicity and self-cleaning properties, due to its coral-like microstructure and low surface energy. The thermal stability experiment of SMA shows that the contact angle is still above 150° when the temperature rises to 190 °C. When the heating temperature is 220 °C, the surface of magnesium alloy changes to the superhydrophobic state. After being soaked in ethanol stearic acid mixed solution, the sample surface is reverted to the superhydrophobic state. These results indicate the SMA has thermal stability and reusability. The SMA sample exhibits a lower corrosion rate compared with the MA sample, revealing that it possessed excellent corrosion resistance. To sum up, this experiment is simple and environment-friendly. It does not require highly polluting reagent, expensive equipment and materials. With the excellent self-cleaning, anti-corrosion, thermal stability and durability, the obtained SMA will have more extensive application and development prospects.

## Figures and Tables

**Table 1:** Electrochemical dates of the polarization curves obtained from the samples.

Sample	E_corr_ (V vs. Ag/AgCl)	I_corr_ (A/cm^2^)	η (%)
AZ91D	−1.2525	3.1713 × 10^−4^	——
SMA	−1.1495	9.7053 × 10^−6^	96.94
